# Optimising Post-Delivery Viral Load Monitoring and HIV Care to Eliminate Breastfeeding-Associated HIV Transmission in Sub-Saharan Africa: A Review

**DOI:** 10.3390/tropicalmed11070202

**Published:** 2026-07-18

**Authors:** Joycelyn A. Dame, Yemah Bockarie, Adraina N. M. Asante, Anthony Enimil

**Affiliations:** 1 Department of Child Health, University of Ghana Medical School, College of Health Sciences, Accra P.O. Box 4236, Ghana; 2 Department of Child Health, Cape Coast Teaching Hospital, Cape Coast P.O. Box CT 136, Ghana; yemahbockarie@yahoo.com; 3 Department of Child Health, Greater Accra Regional Hospital, Accra P.O. Box GP 473, Ghana; amorkorm@yahoo.com; 4 Department of Child Health, School of Medical Sciences, Kwame Nkrumah University of Science and Technology, Kumasi P.O. Box 1934, Ghana; tonash@gmail.com

**Keywords:** post-delivery HIV care, viral load monitoring, vertical transmission, breastfeeding, low-level viraemia, Sub-Saharan Africa, HIV elimination

## Abstract

Sustained maternal HIV viral-load (VL) suppression during breastfeeding is essential for eliminating vertical HIV transmission in Sub-Saharan Africa. Although vertical transmission prevention programmes have improved antiretroviral therapy coverage and peripartum outcomes, viral control may deteriorate after delivery. This decline may result from psychosocial stressors, fragmented mother–infant services, and delayed return of results. This review examines post-delivery VL dynamics, breastfeeding-associated transmission risk, and the limitations of binary suppression thresholds. We suggest that post-delivery VL monitoring should be implemented as a rapid-action pathway. In this approach, detectable viraemia during breastfeeding should prompt a timely reassessment of adherence and repeat VL testing. Regimen review and additional measures to reduce the risks for both the mother and infant should be implemented where indicated. We also examine the individual, relational, structural, and health system determinants of post-delivery viraemia and lost to follow-up, including intimate partner violence. Finally, we highlight service delivery innovations that may improve maternal VL suppression and HIV-free infant survival rates.

## 1. Introduction

The scale-up of antiretroviral therapy (ART) and prevention of vertical transmission (PVT) programmes has markedly reduced paediatric HIV infections in high-burden settings [1,2]. Dolutegravir (DTG)-based regimens are effective, have a high barrier to resistance, and are recommended for pregnant and breastfeeding women [2]. Thus, these advances have shifted the remaining challenge from ART initiation alone to sustained maternal viral suppression throughout the breastfeeding period [3,4]. In Sub-Saharan Africa (SSA), the post-delivery period is a critical point of vulnerability. After delivery, women transition to postnatal, infant, and HIV treatment services. During this period, clinic attendance may decline as childcare demands increase, and financial, social, and emotional challenges may further affect medication adherence and retention in care [5]. Maternal plasma viral load (VL) is the strongest predictor of vertical transmission (VT) risk [6], and viraemia after delivery has direct implications for HIV exposure during the breastfeeding period. Therefore, post-delivery VL monitoring should be considered an important clinical prevention strategy. A detectable VL result during breastfeeding should prompt timely adherence assessment, repeat testing, regimen review where indicated, and maternal–infant risk-reduction measures [7]. However, in many SSA settings, infrequent VL testing and delayed results remain challenges. When clinical action is triggered only at a VL > 1000 copies/mL, clinically relevant low-level viraemia (LLV) below this threshold may be overlooked.

In this review, we highlight the role of post-delivery VL monitoring as a practical strategy for preventing breastfeeding-associated HIV transmission in SSA. We also synthesise evidence on post-delivery VL dynamics and the relationship between maternal viraemia and transmission risk during breastfeeding. We then examine the individual, relational, structural, and health system factors that contribute to viraemia and attrition from care. Finally, we consider monitoring strategies and care models that can sustain VL suppression during breastfeeding. Table 1 summarises the available evidence on the determinants of post-delivery HIV viraemia, their implications during breastfeeding, and the appropriate programme response.

### Search Strategy and Selection Criteria

A targeted literature search was conducted using PubMed/MEDLINE, Google Scholar, the Cochrane Library, and major international HIV guideline repositories to identify relevant studies. English-language publications from January 2010 to June 2026 on post-delivery HIV VL monitoring, breastfeeding-associated HIV transmission, maternal virologic outcomes, adherence, retention, and service delivery were identified. Eligible evidence included primary studies, systematic reviews, implementation studies, and clinical guidelines. Evidence was synthesised narratively without a formal risk-of-bias assessment, focusing on post-delivery virologic vulnerability, breastfeeding transmission risk, determinants of viraemia, lost to follow-up, and strategies to improve VL monitoring and care.

## 2. Post-Delivery Virologic Vulnerability: Timing, Patterns and Implications

### 2.1. Suppression During Pregnancy Does Not Guarantee Suppression During Breastfeeding

Viral suppression achieved during pregnancy may not persist during breastfeeding. During antenatal care, frequent clinic contact, structured PVT counselling, and intensified follow-up can support adherence and viral suppression [2]. After delivery, this support often becomes less consistent as women transition to postnatal, infant, and routine ART services. A prospective Ghanaian cohort illustrates this pattern: high VL suppression in late pregnancy and the early post-delivery period declined substantially by 12 months, alongside considerable attrition [8]. Similar findings from other SSA settings show that disengagement from care and virologic rebound remain common after delivery, despite successful antenatal ART initiation [9,10]. Failure to follow up may lead to prolonged treatment interruption, allowing maternal viraemia to persist undetected during breastfeeding. Women who initiate ART during pregnancy are not the only ones at risk. Those already on treatment may also develop viral rebound due to poor adherence, psychosocial challenges, fragmented care, or interruptions in ART access [11]. These findings show that viral suppression achieved during pregnancy cannot be assumed to continue throughout breastfeeding.

### 2.2. Early Post-Delivery Viraemia Can Predict Longer-Term Non-Suppression

We suggest that early detectable viraemia after delivery should be interpreted as a warning sign of later virologic instability rather than a minor laboratory abnormality. This recommendation is supported by evidence from a Malawian cohort, where women with detectable VL within 1–6 months post-delivery had higher odds of persistent non-suppression later at follow-up [12]. This pattern is consistent with broader evidence that post-delivery rebound often reflects unresolved adherence challenges, treatment interruption, psychosocial strain, or emerging resistance rather than isolated short-term fluctuations [13]. Early post-delivery viraemia should therefore be regarded as an opportunity for intervention rather than reassurance, enabling prompt action to prevent sustained viraemia and reduce breastfeeding-associated HIV transmission.

### 2.3. Distinguishing Transient Blips, Sustained Low-Level Viraemia, and Virologic Rebound: Moving Beyond Binary Thresholds

Current guidelines primarily distinguish virologic suppression from virologic failure using VL thresholds, with the World Health Organization (WHO) defining virologic failure as a VL > 1000 copies/mL after adherence support [2]. Post-delivery viraemia may be interpreted according to three clinically relevant patterns: transient blips, sustained LLV, and high-level virologic rebound. A transient blip is usually an isolated, low-detectable VL result followed by prompt resuppression without major clinical intervention [14]. Such results may reflect assay variability, brief adherence lapses, intercurrent infection, or vaccination and are not equivalent to virologic failure [15]. In contrast, sustained LLV refers to repeated detectable VLs below conventional failure thresholds, often 50–999 copies/mL under WHO definitions [2], although some high-income settings use narrower definitions [15]. Higher-level rebound, or unsuppressed VL, commonly operationalised as a VL of more than 1000 copies/mL, is more strongly associated with prolonged treatment interruption, substantial adherence disruption, pharmacologic problems, or resistance, and requires urgent clinical action [16].

Persistent or rising viraemia is more concerning than a single isolated blip, especially during breastfeeding, when infant exposure accumulates over time. Reviews of LLV indicate that persistent low-level replication or non-suppressible viraemia can predict subsequent virologic failure and drug resistance, particularly when the VL remains above 200 copies/mL or recurs on serial testing [12,17,18]. We suggest that distinguishing these patterns is important during breastfeeding because the risk of HIV transmission and the required clinical response differ across them. Threshold-based responses, using a cutoff of 1000 copies/mL, may miss this risk, particularly in infants who are breastfed for prolonged periods.

## 3. Maternal VL and Breastfeeding Transmission Risk

### 3.1. Maternal VL Is the Strongest Proximal Predictor of Vertical Transmission

The relationship between high maternal plasma VL and VT is well established during pregnancy, delivery, and the breastfeeding period [3,6,19]. Maternal ART and infant prophylaxis substantially reduce transmission risk, but their effectiveness depends on sustained maternal viral suppression throughout pregnancy, delivery, and breastfeeding [6,20]. The principle of “undetectable = untransmittable” (U=U), which is well established for sexual HIV transmission [21], is applied more cautiously to breastfeeding. Breast milk contains both cell-free HIV RNA and cell-associated virus, and local viral shedding may occur independently of plasma VL, contributing to the small residual risk of HIV transmission despite suppressed maternal plasma VL [6,22]. Furthermore, though maternal plasma VL and breastmilk viral activity are closely related, they are not equivalent measures of transmission risk [23]. Uncertainty remains regarding the contribution of cell-associated viruses, local breast inflammation, and the potential impact of short-lived LLV on infant HIV acquisition [24,25]. Findings from a recent meta-analysis suggest that sustained maternal viral suppression, particularly below 50 copies/mL, is associated with very low postnatal transmission risk, while increasing VLs are associated with higher transmission risk [6]. This information is especially relevant in SSA settings, where intermittent testing, delayed result return, mobility, and adherence challenges may allow short episodes of rebound to go unnoticed.

### 3.2. Implications for Infant Prophylaxis

Detectable maternal viraemia during breastfeeding has important implications for infant prophylaxis. The WHO guidelines on prevention of HIV VT recommend enhanced infant prophylaxis for breastfeeding infants of mothers with detectable viraemia and advise continuing prophylaxis until maternal viral suppression is achieved [26]. However, these guidelines do not explicitly define the VL threshold for suppression in this context. WHO treatment guidelines have traditionally used a VL threshold of ≤1000 copies/mL to define virologic suppression for programmatic decision-making [27]. In contrast, the South African guidelines recommend enhanced infant prophylaxis when the maternal VL exceeds 50 copies/mL during breastfeeding, reflecting a more cautious approach to infant risk [28]. This difference raises an important question: Is a programmatic threshold of 1000 copies/mL sufficiently protective during breastfeeding? Emerging evidence suggests that HIV transmission is not restricted to VLs above this threshold and may occur in the presence of persistent or recurrent low-level viraemia, particularly when accompanied by recent treatment interruption, suboptimal adherence, or factors that increase viral shedding in breast milk [3,8,18]. We suggest that decisions on enhanced infant prophylaxis should consider not only VL thresholds but also persistent LLV and the overall clinical context. This highlights the importance of post-delivery VL monitoring to support timely clinical decisions and reduce the risk of HIV transmission during breastfeeding.

## 4. Determinants of Post-Delivery Viraemia and Loss to Follow-Up

### 4.1. Interacting Determinants of Post-Delivery Viraemia and Disengagement from Care

Post-delivery VL rebound and loss to follow-up often result from multiple overlapping challenges. Following childbirth, women must maintain lifelong ART while balancing physical recovery, infant care, household responsibilities and economic demands. These competing demands have been shown to reduce adherence and retention in care [29,30]. Additional barriers include psychosocial stressors, fragmented health services that require transfers after delivery, and inadequate counselling on the importance of maintaining viral suppression during breastfeeding [4,31]. Post-delivery disengagement from HIV care may be influenced by stigma, fear of HIV status disclosure, and intimate partner violence (IPV), which can undermine medication adherence and clinic attendance [32,33]. Perinatal mental health is another important factor, as post-partum depression and anxiety are associated with poor adherence, reduced retention, and difficulties navigating care [34,35]. Therefore, post-delivery viraemia should be understood as the downstream manifestation of accumulated vulnerabilities rather than a simple marker of individual nonadherence.

### 4.2. Care Fragmentation and Health-System Delays May Amplify Post-Delivery Risk

Health system factors may also increase the post-delivery risk. Evidence suggests that fragmented maternal and infant services and transitions from PVT clinics to routine HIV care can increase clinic visit burden, disrupt continuity of care, and increase the likelihood of disengagement from care [36,37]. Operational weaknesses in VL monitoring can further increase post-delivery vulnerabilities. Infrequent testing, delayed results, weak patient tracking, workforce shortages, and ART stock disruptions can all limit the timely identification and management of women at risk of viral rebound [38]. Point-of-care (POC) or near-POC VL testing, rapid result-return mechanisms, and digital follow-up can shorten time-to-action, but only when embedded within integrated care pathways rather than being used as standalone technologies [39]. Thus, the key metric is not VL coverage alone, but whether systems detect post-delivery viraemia early enough and act quickly enough to improve maternal and infant outcomes.

## 5. Optimising Post-Delivery Viral Load Monitoring: From Coverage to Time-to-Action

### 5.1. Timing and Intensity of Post-Delivery Viral Load Monitoring Should Reflect Breastfeeding Risk

Emerging evidence suggests that the risk of viral rebound during breastfeeding is dynamic and that VL monitoring should extend beyond delivery, with closer follow-up for women at increased risk of viraemia [40,41]. Current differentiated service delivery models also recognise that pregnant and breastfeeding women require more intensive monitoring than stable non-pregnant adults because of their unique virologic vulnerability [41].

A risk-stratified approach to post-delivery VL monitoring should align the timing and intensity of testing with periods of greatest breastfeeding-related risk, rather than relying solely on fixed administrative schedules. Early post-delivery VL testing should, where feasible, coincide with the first postnatal or infant follow-up visit to facilitate the early detection of viral rebound before prolonged breastfeeding exposure. Monitoring should continue throughout breastfeeding, with more frequent testing for women with detectable VL, recent ART initiation or regimen changes, adherence issues, and psychosocial challenges. We further suggest that any detectable VL should trigger predefined clinical actions, including repeat VL testing, adherence assessment, and appropriate maternal and infant interventions [4].

### 5.2. Optimising the Viral Load Cascade: From Testing to Timely Clinical Action

Post-delivery VL monitoring has limited preventive value if the results are returned too late to influence maternal management or infant prophylaxis. Delays anywhere along the VL cascade, from specimen collection and laboratory processing to result reporting and clinical action, can allow maternal viraemia to persist undetected during breastfeeding [42]. Electronic result delivery and tracking systems have been shown to improve the timeliness of communication and follow-up within maternal–infant HIV programmes, facilitating earlier clinical action [43]. POC and near-POC VL platforms offer an opportunity to shorten the interval between testing and interventions. Implementation studies from Uganda and Kenya have demonstrated that these approaches are feasible, acceptable, and capable of reducing turnaround times, thereby supporting earlier counselling and clinical decision-making [44,45]. However, successful implementation depends on reliable cartridge supply, equipment maintenance, quality assurance, staff training, and clear referral pathways.

In many SSA settings, a hybrid model combining routine centralised VL testing with targeted near-POC testing for women at the highest risk of viral rebound may offer a pragmatic approach to accelerating maternal management, infant prophylaxis decisions, and other interventions during breastfeeding.

## 6. Post-Delivery HIV Care Models: Integrating Services to Sustain Suppression

### 6.1. Integrated Mother–Infant Care Should Be the Organising Principle of Post-Delivery HIV Services

Integrated mother–infant care can improve continuity of care during the post-delivery period by reducing the fragmentation that often occurs when women transition from PVT services to routine HIV care [46]. Models that align maternal ART refills, VL monitoring, infant immunisation, growth monitoring, and early infant diagnosis are associated with reduced visit burden, improved retention, and more timely maternal–infant risk assessment when viraemia is detected [46,47,48]. Effective programmes go beyond service co-location by coordinating appointments, using shared mother–infant records and tracking systems, and delivering counselling centred on the mother–infant dyad [47]. Coordinated appointments, shared tracking systems, and aligned maternal and infant services should be prioritised to reduce fragmentation, strengthen continuity of care, and facilitate timely responses to maternal viraemia.

### 6.2. Adherence, Psychosocial, and Digital Support Should Be Embedded as Routine Post-Delivery Care

Adherence support should be a routine component of HIV care after delivery. Behavioural and peer-support interventions, including mentor-mother programmes and structured counselling, can improve retention, adherence, and virologic outcomes [49,50,51]. Psychosocial support should be integrated into routine post-delivery HIV care. Screening for IPV, stigma, and perinatal depression, with appropriate referral for support, may improve adherence and retention in care [52]. Digital tools can extend these supports by sending appointment reminders, tracking missed visits, issuing alerts for overdue VL testing, and enabling faster communication of actionable results to care teams [53]. However, they must be designed with strict attention to privacy and disclosure risks, especially when phone access is shared or monitored. Evidence increasingly supports multicomponent care packages that combine peer support, psychosocial assessment, differentiated service delivery, and secure digital follow-up to improve retention in care and sustain maternal viral suppression during breastfeeding [41].

## 7. Limitations

As a narrative review, we did not conduct a formal risk-of-bias assessment or meta-analysis, and the selected literature may influence the synthesis. Evidence on post-delivery low-level viraemia remains limited and heterogeneous, with variations in VL thresholds, testing intervals, and definitions of transient blips, persistent LLV, and virologic rebound. In addition, few contemporary breastfeeding cohorts have quantified the transmission risk across low but detectable VL ranges, particularly among women receiving DTG-based ART. Because much of the available evidence comes from selected programmes or cohort settings, the findings may not be fully generalisable across diverse SSA health systems.

### Conclusions

Eliminating HIV VT in SSA requires not only high antenatal ART coverage but also sustained maternal viral suppression throughout the breastfeeding period. Evidence shows that viral rebound and loss to follow-up remain important threats to HIV-free infant survival. Therefore, programmes should treat VL monitoring as a rapid action pathway, linking detectable viraemia to timely adherence support, clinical review, infant risk assessment, and breastfeeding counselling. Integrated mother–infant care and coordinated response systems are essential to prevent transient viraemia from becoming a sustained risk for both the mother and child.

## 8. Future Directions

Future research should define the risk of breastfeeding-related HIV transmission at different maternal VL levels in the era of DTG use. It should also identify the drivers of post-delivery disengagement from care. Finally, implementation studies should evaluate integrated mother–infant care, peer support, digital follow-up, and POC VL testing to improve retention, sustain viral suppression, and achieve HIV-free survival rates in infants. These findings will inform scalable strategies to eliminate breastfeeding-associated HIV transmission in SSA.

## Figures and Tables

**Table 1:** Determinants of post-delivery viraemia during breastfeeding, associated risks, and appropriate programme responses.

Domain	Key Determinants	Breastfeeding Risk	Programme Response
Individual/clinical	Poor adherence, treatment interruption, adverse effects, detectable post-delivery VL, depression, anxiety	Viral rebound, low-level viraemia, prolonged infant exposure	Early and repeat VL testing, adherence assessment, regimen review, and mental health support
Psychosocial	Stigma, nondisclosure, limited social support, intimate partner violence, and conflicting feeding advice	Poor adherence, missed visits, and delayed risk identification	Confidential counselling, intimate partner violence support, peer/mentor-mother programme
Structural/economic	Transport costs, relocation, work and childcare demands, food insecurity	Missed appointments, delayed VL testing, and ART interruption	Differentiated care, social support
Health system	Fragmented services, infrequent VL testing, delayed results, weak follow-up systems	Late detection of viraemia, delayed action, and increased infant exposure	Integrated mother–infant visits, rapid result delivery, active recall, targeted point-of-care VL testing

## Data Availability

No new data were created or analyzed in this study. Data sharing is not applicable to this article.
